# The Disease Portals, disease–gene annotation and the RGD disease ontology at the Rat Genome Database

**DOI:** 10.1093/database/baw034

**Published:** 2016-03-23

**Authors:** G. Thomas Hayman, Stanley J. F. Laulederkind, Jennifer R. Smith, Shur-Jen Wang, Victoria Petri, Rajni Nigam, Marek Tutaj, Jeff De Pons, Melinda R. Dwinell, Mary Shimoyama

**Affiliations:** ^1^Medical College of Wisconsin, Human and Molecular Genetics Center; ^2^Department of Physiology, Medical College of Wisconsin; ^3^Department of Surgery, Medical College of Wisconsin, Milwaukee, WI, USA

## Abstract

The Rat Genome Database (RGD; http://rgd.mcw.edu/) provides critical datasets and software tools to a diverse community of rat and non-rat researchers worldwide. To meet the needs of the many users whose research is disease oriented, RGD has created a series of Disease Portals and has prioritized its curation efforts on the datasets important to understanding the mechanisms of various diseases. Gene-disease relationships for three species, rat, human and mouse, are annotated to capture biomarkers, genetic associations, molecular mechanisms and therapeutic targets. To generate gene–disease annotations more effectively and in greater detail, RGD initially adopted the MEDIC disease vocabulary from the Comparative Toxicogenomics Database and adapted it for use by expanding this framework with the addition of over 1000 terms to create the RGD Disease Ontology (RDO). The RDO provides the foundation for, at present, 10 comprehensive disease area-related dataset and analysis platforms at RGD, the Disease Portals. Two major disease areas are the focus of data acquisition and curation efforts each year, leading to the release of the related Disease Portals. Collaborative efforts to realize a more robust disease ontology are underway.

**Database URL:** http://rgd.mcw.edu

## Introduction

In response to the observation that rat researchers, unlike researchers using other model organism databases, tend to be more disease oriented (1), the Rat Genome Database (RGD) has directed a significant portion of its efforts toward disease curation. Rat researchers’ interests often revolve around hypertension and diabetes, which are the topics of two of the earliest RGD disease portals established to address the needs of these research communities. They often use multiple model organisms and are clinically oriented, which is why RGD makes disease annotations not only from rat disease models, but from human disease and mouse disease models as well, for a fuller picture of the human disease. RGD curators generate gene–disease annotations from the scientific literature using in-house-designed curation software (2). The use of a controlled disease vocabulary in this process helps maintain data consistency and assists in data analysis. RGD, which started in 1999 curating genes primarily to the Gene Ontology (GO) (3) and Mammalian Phenotype Ontology (4), began generating disease annotations shortly thereafter using disease terms from the C branch of the Medical Subject Headings (MeSH) (5). As pointed out in detail by Davis *et al.* (6) disease ontologies and vocabularies such as MeSH, Online Mendelian Inheritance in Man (OMIM) (7), the Disease Ontology (8, 9), Systematized Nomenclature of Medicine-Clinical Terms (SNOMED-CT; http://www.ihtsdo.org/SNOMED-CT/) and the Unified Medical Language System (UMLS) Metathesaurus (10) are all individually less than ideal due to issues of stability, maturity, public availability and ready accessibility. To address these problems, Davis *et al.* at the Comparative Toxicogenomics Database (CTD, http://ctdbase.org/) developed MEDIC, a combined vocabulary merging OMIM terms into the MeSH disease term hierarchy. As they point out, MEDIC is technically not an ontology—it has less formally constrained, less well-defined inter-relationships between terms than an ontology. In 2012, in the absence of a stable, available disease ontology that suited our needs, RGD adopted MEDIC as its disease vocabulary and began adding its own terms as needed, calling it the RGD Disease Ontology (RDO).

## The RDO

The RDO is developed using the Open Biomedical Ontology (OBO) format. The vocabulary is edited as needed for completeness. New terms are added to the vocabulary as siblings or children of appropriate existing terms, adding links to corresponding individual pages at MeSH and/or OMIM. The terms added to the RDO by RGD are unique to the RDO and are not present in MEDIC. The vocabulary is updated with new RGD-generated terms periodically as needed, and with any new MEDIC terms weekly via a pipeline from CTD. It is freely available for download and use in OBO format at the RGD ftp site (ftp://ftp.rgd.mcw.edu/pub/ontology/).

Recently, editing of the RDO has been added to the functionality of the RGD curation software. New terms are added and edited in the Object Edit tool, the same tool that allows additions of strains, quantitative trait loci (QTLs) and genes. Synonyms, identifiers, external database references and term relationships can all be added or deleted from an RDO entry. The Object Edit tool can be accessed directly from the curation version of the RGD term browser, so the combination of the editor and browser performs similarly to a dedicated ontology editing tool.

The quality control effort for the RDO involves collaboration with curators at CTD. If RGD curators find any discrepancy with terms in MEDIC, they are sent to CTD for resolution. As of January 2016, the RDO had >12 900 terms. Since the adoption of MEDIC by RGD, the number of terms that have been added by RGD is over 1000. The number of new terms added to the vocabulary averages ∼30 per month.

## Manual disease curation at RGD

**Figure 1.**
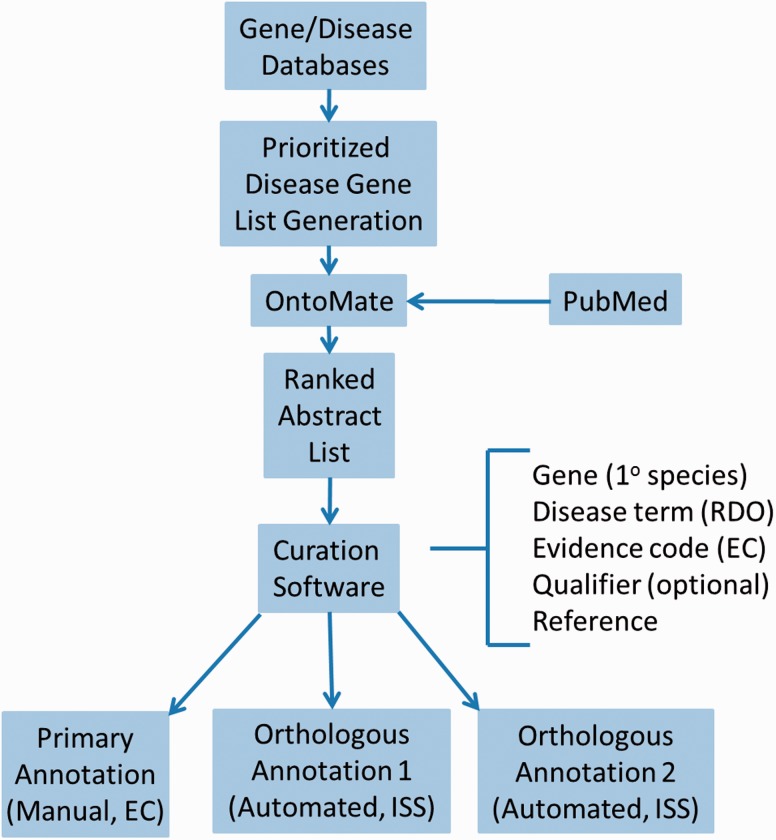
Disease curation flow diagram. Prioritized gene lists derived from disease databases are used to search PubMed using the text mining tool OntoMate. The ranked abstract list obtained is manually curated to generate primary and orthologous annotations.

For efficient and comprehensive coverage, disease curation at RGD involves working from a prioritized gene list targeted at a particular disease area, usually the subject of a Disease Portal in development. To generate such lists, all applicable adjectives, synonyms and sub-types of relevant diseases are assembled from MeSH. Disease definitions from at least two other sources are consulted for any synonyms, adjectives or sub-types not present in MeSH. These terms should be specific for the disease in question. At least three gene/disease databases are queried with the assembled terms, such as Phenopedia (https://phgkb.cdc.gov/HuGENavigator/startPagePhenoPedia.do), GeneCards (http://www.genecards.org), Genetic Association Database (http://geneticassociationdb.nih.gov) and Genatlas (http://genatlas.medecine.univ-paris5.fr/imagine/home.php). Databases dedicated to the disease in question are located with internet searches. Retrieved gene lists from all sources are transferred to a single list, alphabetized by gene name on a spreadsheet, retaining the gene symbol and name, species, source and number of disease-related publications mentioning the gene (“hits”) from each source. Genes are then ranked based on the combined number of “hits” from all sources. Higher-ranking genes are then given priority for curation (Figure 1).

**Figure 2.**
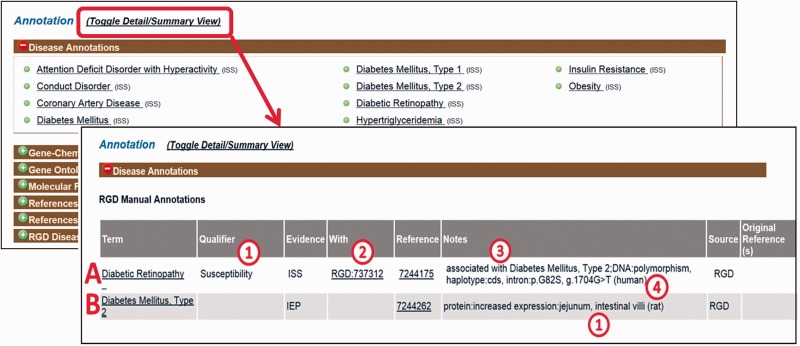
Disease annotation display examples. Shown fields are term, qualifier (A1), evidence code, “With” field (A2), reference, “Notes” field composed of background disease field (A3), alteration level field, modifier field, location field (two entries, B1), free text field (A4).

First, PubMed abstracts are searched using the text mining software tool OntoMate (11) which has been integrated into the curation software (2). The search retrieves and ranks abstracts containing selected disease terms and the name and synonyms of selected genes (Figure 1). To make a disease annotation, a curator links gene objects and a literature reference with a RDO disease term and a manual annotation evidence code (12) indicative of the relationship of the gene to the disease. The evidence code Inferred from Expression Pattern (IEP) signifies a biomarker for the disease. The code Inferred from Phenotype Manipulation (IPM) is used in cases where gene expression is artificially altered and a genetic or mechanistic connection between the gene and disease is implied. Evidence of involvement in the molecular mechanism of the disease or a pathway known to contribute to a disease state is indicated with the evidence code Inferred from Experimental Data (IED). For an association of the disease with one or more genetic mutations or polymorphisms, the Inferred by Association of Genotype from Phenotype (IAGP) code is used. Qualifiers can be added to indicate whether the gene is associated with disease onset, severity, progression or susceptibility, is involved with or responsive to treatment or is not genetically associated with the disease. Primary annotations are manually made to one of the three species, rat, human or mouse, and the curation software automatically generates annotations for the orthologous genes in the other two species (Figure 1) using the evidence code Inferred from Sequence or Structural Similarity (ISS), with the Gene ID from the primary species identified in the “With” field (Figure 2A-2). Figure 2 shows the more detailed annotation view obtained by clicking “Toggle Detail/Summary View” at the top of the “Annotation” section of a gene report page. As of January 2016, RGD possessed over 30 600 manual and 60 100 computationally inferred disease annotations generated in-house. These are shown broken down into the different evidence codes in Table 1.

**Table 1.** Categorization of disease data

Source	Evidence code	Annotations
Manual gene–disease annotations		
RGD	IAGP	8381
RGD	IED	3913
RGD	IPM	4343
RGD	IEP	16 290
Computationally inferred gene–disease annotations		
RGD	ISS	64 532
Imported computational gene–disease annotations		
ClinVar	IEA	80 548
OMIM	IEA	5702
GAD	IEA	222
Imported inferred gene–disease annotations		
ClinVar	ISS	47 946
OMIM	ISS	9554
GAD	ISS	397
Total		241 828

**Figure 3.**
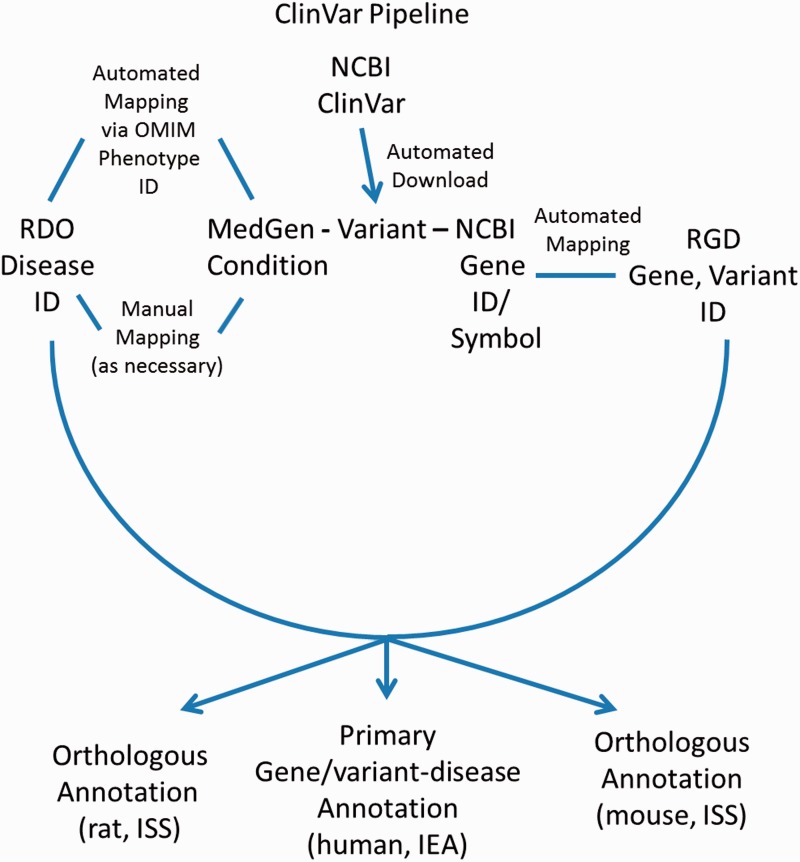
ClinVar Automated Import and Annotation Pipeline flow chart.

**Figure 4.**
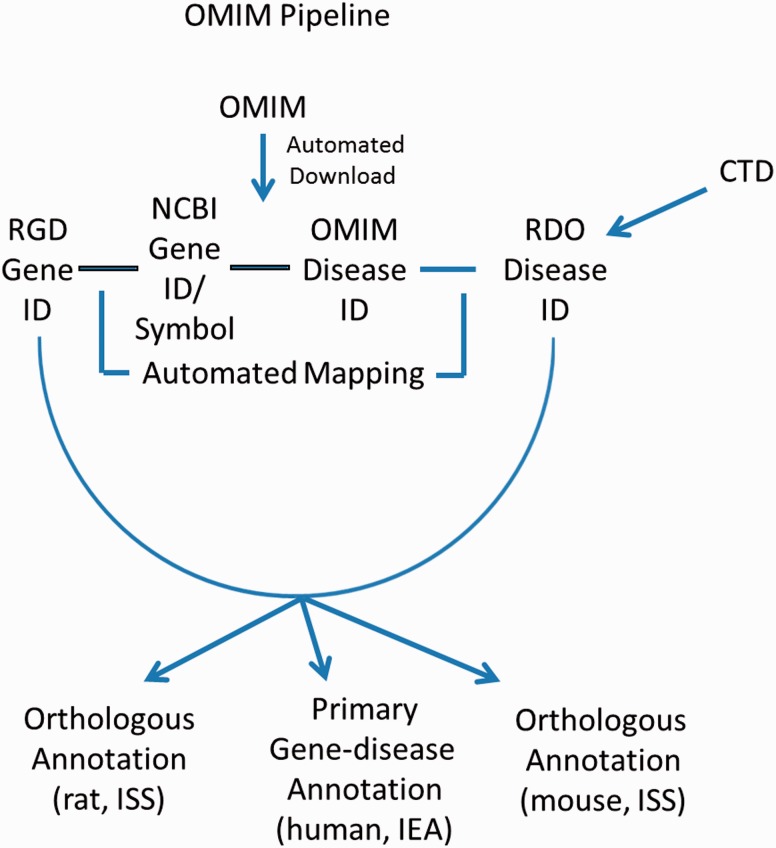
OMIM Disease Annotation Pipeline flow chart.

For IEP and IAGP annotations, a set of structured notes fields are used to provide additional information (Figure 2A-3). Fields within the notes are separated by colons to facilitate parsing. If a background disease is present, this is indicated in an optional field at the beginning of the note (Figure 2A-3) which is separated from the rest of the fields by a semicolon. When a background disease is not present, in the first field, the level of the alteration is specified (DNA, mRNA or protein). In the second field, a modifier is provided indicating the type of alteration, taken from standardized sets of terms for each level. The third field indicates the anatomical site, cell type, cell component or sequence location using the Uberon (13), Cell (14), GO cellular component (3) and Sequence (15) ontologies, respectively. A fourth free text field is for specific mutated base or sequence standardized nomenclature (16) or other information such as dbSNP identifiers or species which are given in parentheses (Figure 2A-4). For any single annotation, a field can contain up to three entries (Figure 2B-1); for more than three, “multiple” is specified.

## Disease annotation pipelines

### ClinVar

In addition to disease annotations manually generated at RGD, a series of automated pipelines brings in annotations from outside sources. Disease annotations from the National Center for Biotechnology Information’s (NCBI) ClinVar database (http://www.ncbi.nlm.nih.gov/clinvar/) (17) are brought in to RGD by the ClinVar Automated Import and Annotation Pipeline (Figure 3). Variants with assigned MedGen (http://www.ncbi.nlm.nih.gov/medgen/) (18) conditions and gene associations are downloaded from NCBI's ClinVar FTP site at ftp://ftp.ncbi.nlm.nih.gov/pub/clinvar/xml/ClinVarFullRelease_00-latest.xml.gz. Imported variants are associated with RGD genes based on the NCBI Gene ID or gene symbol. The ClinVar FTP file (ftp://ftp.ncbi.nlm.nih.gov/pub/clinvar/gene_condition_source_id) maps NCBI MedGen condition IDs (“ConceptID”) to OMIM phenotype IDs. Because the RDO uses OMIM IDs as aliases for disease terms, OMIM IDs from the ClinVar data can be matched in turn to RDO terms, which are then assigned to RGD variant and gene records as RDO annotations. For MedGen terms that do not map to OMIM IDs, the RGD curators manually map the terms to pre-existing RDO terms, or create new RDO terms. Annotations generated from ClinVar are given an evidence code of Inferred from Electronic Annotation (IEA) for human (the source species) and are automatically propagated to the orthologous rat and mouse genes with the ISS evidence code (Figure 3). The source of all these annotations is listed as “ClinVar”. The annotations are displayed on both the RGD variant report page and the associated RGD gene report page.

### OMIM

These gene–disease annotations are created and brought in to RGD by the OMIM Disease Annotation Pipeline (Figure 4). The OMIM pipeline associates OMIM disease IDs with RGD genes via the NCBI Gene ID. This pipeline uses data imported from CTD and existing OMIM-ID-to-RGD-gene associations imported from NCBI’s Gene database to make RDO annotations. Since data in OMIM are human data, and since these data were not reviewed by RGD curators before making assignments, the evidence code assigned to human gene–disease annotations is IEA. Annotations are also propagated to the orthologous rat and mouse genes based on the similarity of the respective sequences. To reflect this, the evidence code for the mouse and rat annotations is ISS (Figure 4). The pipeline is run and annotations updated on a weekly basis.

### Genetic Association Database

Disease annotations were imported into RGD from the National Institute of Health’s Genetic Association Database (GAD) (19). These annotations are given an evidence code IEA for human (the source species) and are automatically propagated to the orthologous rat and mouse genes with the evidence code ISS. As of September 2014, the GAD has been retired, although the data are still accessible by download at http://geneticassociationdb.nih.gov, and are maintained at RGD as a legacy dataset that is no longer updated. RGD hosted over 138 900 imported disease annotations from the three databases as of January 2016, shown by database and evidence code in Table 1.

## The Disease Portals

**Figure 5.**
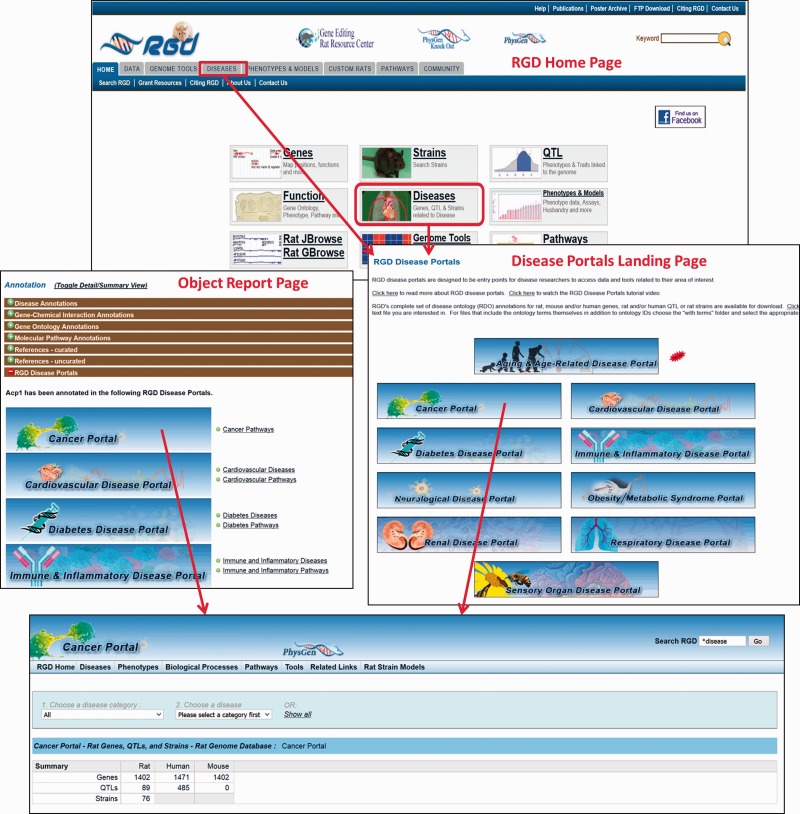
The RGD home page showing Disease Portal entry points. Disease Portals can be accessed via the landing page or object report pages.

The Rat Genome Database’s Disease Portals are an integrated resource for information on genes, QTLs and strains associated with a variety of disease conditions. They house diverse datasets—not only genes, but also QTLs, pathways, phenotypes, biological processes and strains. They can be accessed via a landing page from the tab and central panel on RGD’s home page (Figure 5). Alternatively, Disease Portals can be accessed directly from individual object (gene, QTL or rat strain) report pages. Information about the portal(s) in which a given gene, QTL or strain appears is available in the “RGD Disease Portals” portion of the “Annotation” section (Figure 5).

As an example, one of the latest portals, the RGD Sensory Organ Disease Portal, is an integrated resource of data related to sensory organ diseases. Terms for sensory organ diseases, or sensory organ disease-related phenotype terms, GO biological process terms specifically related to sensory organ function, development and related pathway terms are selected by curators to populate the portal. The genes, QTLs and strains annotated to those terms are automatically incorporated into the portal in the appropriate section, to point researchers toward genes that are or could be involved in sensory organ disease.

**Figure 6.**
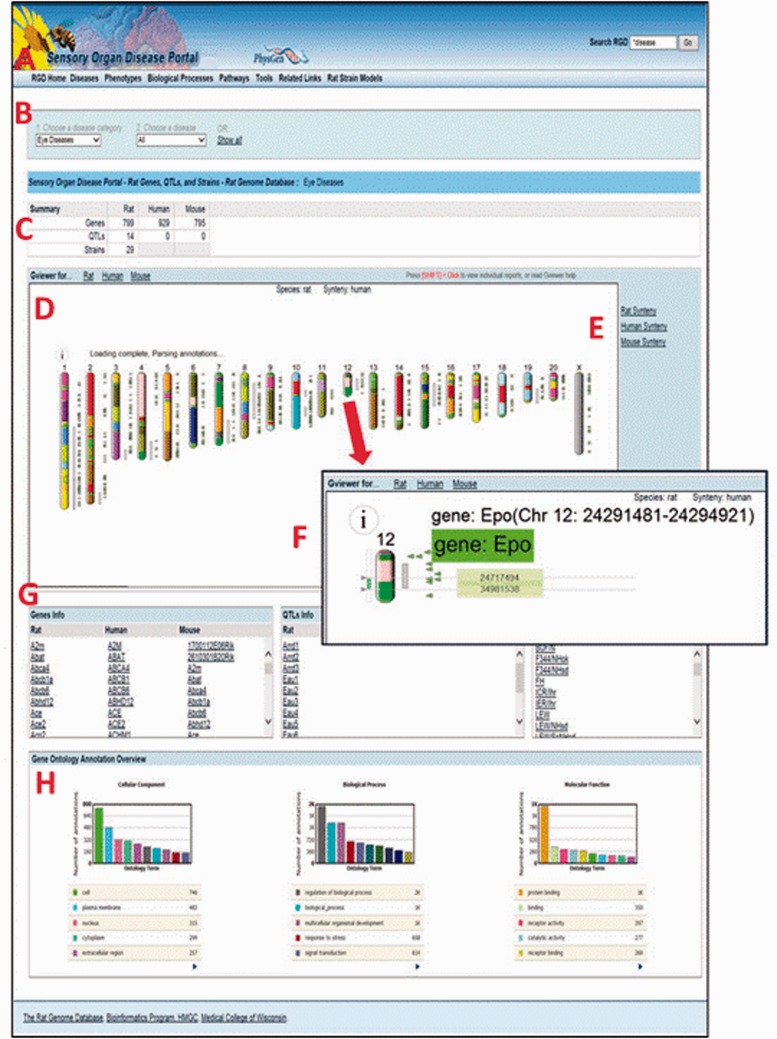
The Sensory Organ Disease Portal main page. **A**, category tabs; **B**, term drop-down menus; **C**, summary table; **D**, Genome Viewer; **E**, synteny function; **F**, single chromosome view and functionality; **G**, gene, QTL, strain lists; **H**, portal GO term annotation frequencies.

All Disease Portals are organized into various tabs, each of which covers one of the incorporated disease-related datasets, i.e., “Diseases”, “Phenotypes”, “Biological Processes” or “Pathways” (Figure 6A). For ease of use, the page format is shared across all four major pages of the Disease Portal. On any of these pages, data can be viewed for all the categories or selections can be refined using drop-down lists of terms.

In any part of the portal, the first drop-down list (Figure 6B) is used to choose a disease, phenotype, biological process or pathway term. The default is presentation of all the data related to that term and its children. To narrow a search further, a more specific term from the second list can be chosen.

For researchers interested in translational research and/or cross-species analysis, RGD’s Disease Portals contain additional data for mouse and human from external databases such as the Mouse Genome Informatics Database (20) and the NCBI (18). In addition, manual disease and pathway annotations, as well as human and imported mouse QTL data are provided by RGD. This allows the user, e.g. to leverage the extensive body of phenotype data for genes and QTLs from mouse knockout studies or to compare the data from rat models against clinical data from human studies. A summary table at the top of the page (Figure 6C) gives a count of the data objects (genes, QTLs or strains) that match the search criteria, grouped by data type and species.

The Genome Viewer (GViewer) tool (Figure 6D) shows all mapped objects against the full set of chromosomes for the selected species. It provides a genome-wide view of the data that match the selected terms. The genes and/or QTLs can be viewed beside the rat, mouse or human chromosomes. Clicking a gene or QTL symbol accesses the corresponding RGD report, giving the user one-click access to a more comprehensive view of the data available for that object, including references. The synteny function (Figure 6E) is used to view a color-coded depiction of the corresponding segments for another species. The corresponding segments of chromosome in another species can be found using the mouse or human synteny selections.

Clicking on a chromosome in the GViewer selects it for a closer view (Figure 6F). Right-clicking zooms in further. The arrows on the left are dragged to select a region and clicking on the selected region links to the GBrowse tool (21) for further exploration. Mousing over an icon provides information about that object.

Below the GViewer are scrollable lists of genes, QTLs and/or strains (Figure 6G) which match the search criteria. QTLs and strains are not annotated with pathway or GO biological process terms, so the lists on pathway or biological process portal pages only contain gene data.

Bar graphs at the bottom of the page show a GO “slim” view of annotations to the displayed list of genes (Figure 6H).

Of the remaining tabs (Figure 6A), “Tools” provides links to a variety of tools for data mining and analysis that can be used to delve further into the information in the portal. Among others, these tools include the Object List Generator and Analyzer (OLGA), a list builder allowing one to compare and contrast object lists based on different criteria. The Gene Annotator tool is employed to explore all of the functional annotations associated with a list of disease-related genes. The rat, mouse and human JBrowse and GBrowse genome browsers can be utilized to view disease-related genes, QTLs and congenic strains in their larger genomic context. The PhenoMiner tool is employed to find quantitative phenotypes for a list of disease-related strains and the Variant Visualizer is used to locate possible disease-causing and strain-specific variants in a region or regions of interest. The “Tools” page provides links to additional ontology analysis tools as well.

“Related Links” contains links within and outside of RGD that provide additional resources for clinical, scientific and bioinformatic information, i.e. general information about one of the sensory organ diseases or phenotypes, a detailed presentation of a disease-related pathway, or what is known about the genetics of one of the diseases in the portal.

“Rat Strain Models” provides easy access to information on rat strains which are demonstrated models for one or more human diseases included in that portal.

**Figure 7.**
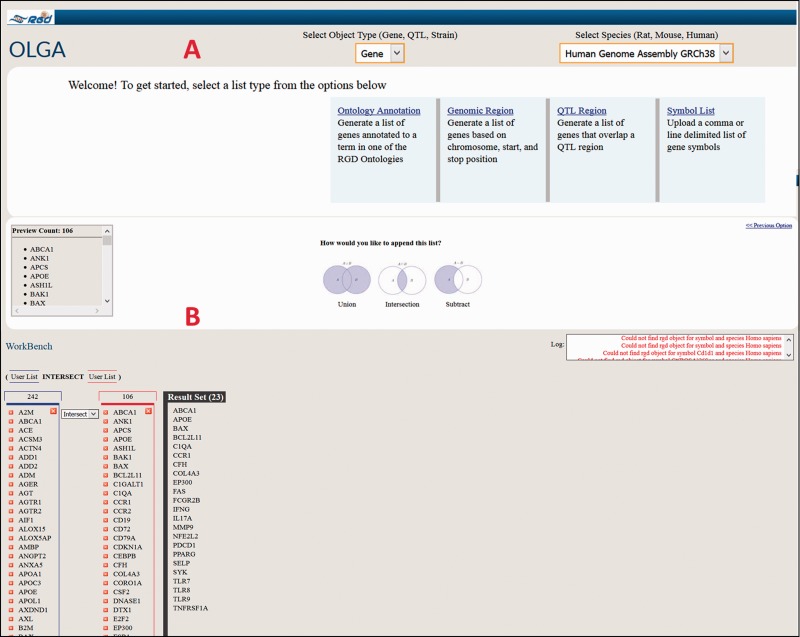
Cross-species use case for identifying genes associated with glomerulonephritis using the Renal Disease Portal and the OLGA tool.

As an illustrative cross-species example of the use of the Disease Portals, one may wish to find which genes are involved in glomerulonephritis. An intuitive way to do this in the Renal Disease Portal might be to select “Nephritis” and “Glomerulonephritis” from the default disease category and disease pull-down menus (Figure 6B), which provides lists of 211 rat, 215 human and 207 mouse genes (Figure 6G) annotated to “Glomerulonephritis” and/or to any of its more specific child terms. To pare the list of genes down to a more manageable number, one might hypothesize that human genes annotated to the disease term “Glomerulonephritis” whose orthologs are annotated to the “glomerulonephritis” phenotype term in another, evolutionarily separated species, such as mouse, would comprise a shorter list of genes more likely to be more centrally involved in the disease. Taking advantage of the substantial mouse phenotype data presented in RGD Disease Portals, one can select the “Phenotypes” tab at the top of the Renal Disease Portal page, followed by phenotype category “kidney inflammation” and phenotype “glomerulonephritis” to obtain the list of mouse genes annotated to that phenotype term. The lists of human disease-annotated genes and mouse phenotype-annotated genes can then be highlighted, copied and pasted to a spreadsheet. The common orthologous genes of these two gene lists, human genes annotated to the disease term “Glomerulonephritis” and mouse genes annotated to the phenotype term “glomerulonephritis”, are then found using the OLGA tool, whose link is in the “Tools” section of all Disease Portals (Figure 6A). In OLGA, “Gene” is selected as “Object Type”, “Human Genome Assembly GRCh38” is selected as “Species” and “Symbol List” is selected as list type (Figure 7A). The human gene list is added, followed by the mouse gene list, with the tool automatically converting the latter list to human orthologs where possible. “Intersection” is selected to generate a third, more manageable list of 22 genes (Figure 7B) for further analysis in which the human ortholog is associated with “Glomerulonephritis” and the mouse ortholog has a demonstrated link to the phenotype “glomerulonephritis”; evidence from two species that these genes might be involved in this disease.

## Future directions

Together with MGI (20) and DO (8, 9) developers, RGD has recently acquired funding to support the continued development and modification of a robust, structured and complete human disease ontology for future use by both databases and the larger research community. This will take the form of a large initial effort involving all three groups over the course of 1 year, including terms suggested by RGD that would be useful, with inferred parentage to make the terms easier to locate. This will be followed by ongoing expansions and improvements.

Leveraging the structure of the disease ontology, a future plan for gene report pages at RGD is to organize disease annotations, which can be numerous for some genes, into expandable lists under higher level disease terms. The more structured organization of the annotations will allow an improved, more navigable presentation of data. The text mining tool OntoMate is being upgraded so that it will scan entire papers rather than just abstracts. The Disease Portals will be revised to have greater connectivity to the tools, including the newly adopted JBrowse, and other data available at RGD, making them true gateways for data mining and analysis beyond the initial retrieval step. Improvements will also include download options for retrieved object lists. Upcoming Disease Portals will include blood diseases and diseases associated with development.

## Funding

This work was supported by the National Heart, Lung and Blood Institute on behalf of the National Institutes of Health [grant numbers HL064541, HL094271]. Funding for open access charge: National Heart, Lung and Blood Institute on behalf of the National Institutes of Health [grant number HL64541].

*Conflict of interest*. None declared.
